# The head fixation based on skull cap: An improved protocol used in single unit recording in the vestibular system

**DOI:** 10.1016/j.mex.2020.101109

**Published:** 2020-10-16

**Authors:** Pengyu Ren, Bowen Li, Shiyao Dong, Boqiang Lyu, Shouping Gong, Qing Zhang, Jianqiang Qu, Peng Han

**Affiliations:** a Department of Neurosurgery, the Second Affiliated Hospital of Xi'an Jiaotong University, 157 West 5th Road, Xi'an, Shaanxi 710004, China; b Departments of Otolaryngology-Head & Neck Surgery, Johns Hopkins University School of Medicine, 720 Rutland Ave, Baltimore, MD 21205, United States; c Division of Health Sciences Informatics, Johns Hopkins University School of Medicine, 2024 E Monument St, Baltimore, MD 21205, United States; d Department of Otolaryngology-Head & Neck Surgery, Xinhua Hospital of Shanghai Jiaotong University, 1665 Kongjiang Rd, Shanghai 200093, China; e Department of Otolaryngology-Head & Neck Surgery, the First Affiliated Hospital of Xi'an Jiaotong University, 227 Yanta West Roud, Xi'an, Shaanxi 710061, China

**Keywords:** Head fixation, Skull cap, Single unit recording, Improved protocol, The vestibular system

## Abstract

Single unit recording has an important application in neuroscience, especially in the vestibular system such as visual stabilization, posture maintenance, spatial orientation and cognition. However, single unit recording conducted in living animals is a demanding technique and non-ideal mechanical stability between the recording location of nerve tissues and the tip of microelectrode always results in failure to obtain successful recordings in the vestibular system. In order to improve the mechanical stability during single unit recording, we constructed a novel head fixation method based on skull cap. This article describes in detail how to construct this novel head fixation. Following the step-by-step procedure mentioned in this article will provide a high-quality mechanical stability for single unit recording in the vestibular system, allowing us to successfully record the nonlinear neural dynamic response over a big magnitude motion stimulation. This improvement of head fixation contributes to the in-depth understanding of the vestibular system.

## Graphical abstract

**Figure:**
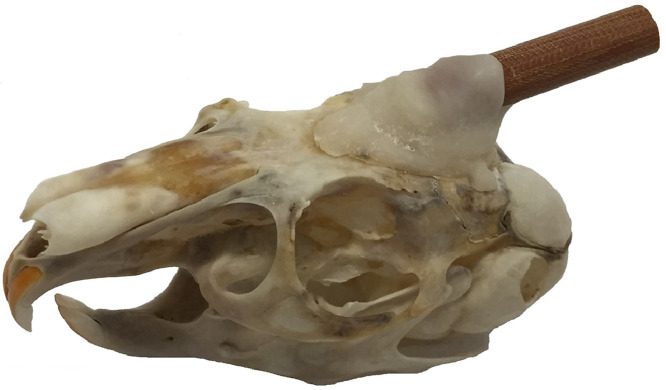

**Specifications table**

**Table:** 

**Subject Area**	*Neuroscience*
**More specific subject area**	*sensory nervous system, vestibular system, neurophysiology*
**Protocol name**	*The protocol of head fixation based on skull cap used for single unit recording*
**Reagents/tools**	*Refer to the main text*
**Experimental design**	*Very brief experimental description*
**Trial registration**	*Not applicable*
**Ethics**	*Animal procedures described in this study are approved by the Animal Care and Use Committee of the Johns Hopkins University School of Medicine and Xi'an Jiaotong University Health Science Center.*
**Value of the Protocol**	• *Non-ideal mechanical stability between the recording location of nerve tissues and the tip of microelectrode always results in failure to obtain successful recordings in the vestibular system.* • *This paper describes a novel head fixation based on skull cap contributing to improve the mechanical stability during single unit recording.* • *This method can help us to successfully record the nonlinear neural dynamic response over a big magnitude motion stimulation, contributing to the in-depth understanding of the vestibular system.*

## Introduction

Single unit recording is a demanding technique, widely used in the investigation of the vestibular system. Besides a good understanding of the technique and equipment required, the most important concern to single unit recording is the mechanical stability between the recording location of nerve tissues and the tip of microelectrode. In previous studies about the vestibular system, the mechanical stability was generally provided by the head fixation based on two ear bars inserting into the left and right external auditory canal and a tube face mask holding the mouth [1], [2], [3]. This head fixation was suitable for single unit recording to record the neural dynamic response data when the magnitude of motion stimulation was small (e.g. head angular velocity <100 deg/s). However, it was no longer qualified to this work because of the extremely high failure rate of data record when the magnitude of motion stimulation was big (e.g. head angular velocity > 180 deg/s) [4]. Our recent study exhibited that the intact dynamic data of semicircular canal afferent neurons in response to the sinusoidal rotation stimulation with an amplitude as big as 300 deg/s, where the mechanical stability for single unit recording was provided by a novel head fixation based on skull cap [5]. These results suggests that the nonlinear neural dynamic data in response to the big magnitude motion stimulation can be accessed through single unit recording with the help of this novel head fixation based on skull cap. In this article, we described in detail how to construct the head fixation base on skull cap in living chinchilla, which can help us to extend the understanding of the vestibular system deeply.

## Experimental procedure

### Ethical statement

All animal procedures are in compliance with the protocol approved by the Animal Care and Use Committee of the Johns Hopkins University School of Medicine and Xi'an Jiaotong University Health Science Center.

####  

**Table:** Materials and instruments

*Materials and instruments*

0.5% xylocaine
0.5% iodophor
BD syringe (5 ml)
Scalpel
Isoflurane (liquid)
Electrocautery
wooden rod (8 mm in diamete)
anesthesia vaporizer
Glass micro pipette (model M1B100F-4)
Kimwipse
Microelectrode puller (P-30)
Sodium Chloride (crystal)
Upright Digital Microscope (LV150N)
Impedance Meters (SYS-OMEGAZ)
PTFE coated silver wire (AG10T)
Animal stereotaxic chair
Ear Bar
Tube face mask
Supporting frame
Gimbaled superstructural platform
Servo-controlled rate motor table (D083M-22–1310)
DC temperature controller (model 40–90–8B)
Otological drill
Cautery (AU-1098)
Surgical Microscope (ZEISS Universal S2)
Medical suction pump (220 VAC S230A)
Universal dental adhesive (model 35,108)
UV curing light (RU1200)
Grip cement powder (675,571)
Grip cement bulk liquid (675,572)
Three-dimensional manipulator (model US-3F)
Hydraulic Microdrive (models MO-22)
Headstage Preamplifier (resistance 1.1 K to 10 K)
Extracellular preamplifier system (2400A)
CED Spike2 neural signal acquisition (Micro1401–3)
Accelerometer (MPU-9250A)
Oscilloscope (DPO2024)
Loudspeaker (Pro-X7)

*Note:*

The animals used in this protocol are chinchillas (*C. laniger*) from 480 to 550 g body weight. Chinchillas are housed in individual cages (one per cage) with free drinking and eating. The environment for chinchilla live is kept at a constant temperature (18–23 °C), a constant humidity (65%) and a 12-hour photoperiod. All animal operations and experiments are performed during the day.

#### Animal anesthesia

**Fig 1:**
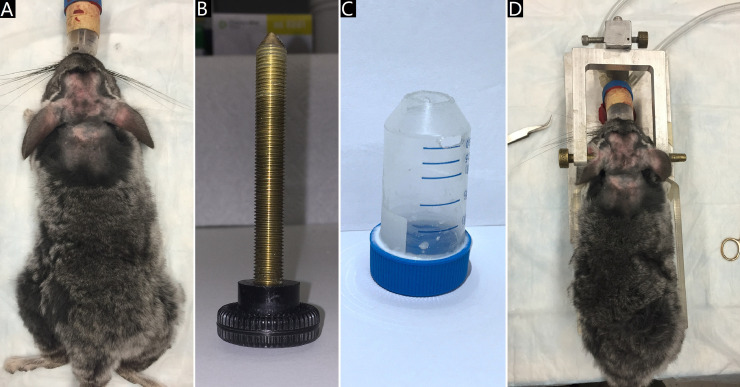
The animal anesthesia and head fixation based on ear bars and a tube face mask. A: animal anesthesia using 1–5% isoflurane; B: the ear bar; C: the tube face mask; D: animal head fixation achieved by two ear bars inserting into the left and right external auditory canal and a tube face mask holding the mouth.

1. Chinchilla is sent to an air-proof box with direct administration of 5% isoflurane for anesthesia induction.

2. Afterwards, Chinchilla is switched to the tube face mask with 2% isoflurane for anesthesia maintenance (Fig 1A).

3. A DC temperature controller (FHC, model 40–90–8B) is used to maintain the core body temperature between 36 and 38 °C.

4. The vital signs of chinchilla are monitored dynamically and assessed every half hour to avoid the harmful level anesthetic accumulation, which can cause abnormal breathing, heart rhythms disorder, low body temperature and even animal death.

*Note:*

Aiming to avoid the harmful level anesthetic accumulation, the does of isoflurane during anesthesia maintenance usually needs to be adjusted according to the animal vital signs. Thus, the concentration of isoflurane used for anesthesia maintenance is fluctuant around 2% (generally between 1% to 3%) in this protocol.

#### Animal head fixation one

5. The hair on the top of the animal head is shaved first to expose the skin.

6. The animal is put into the animal stereotaxic chair.

7. Two ear bars along the head line connecting centers of left and right external auditory canal are inserted into the left and right external auditory canals for preliminary head fixation (Fig 1B-D).

8. A tube face mask along the longitudinal axis of animal body is used for mouth fixation, in which the nasal bone and incisive bone are pushed to make sure that the incisor teeth are fixed on the mask side wall [5] (Fig 1B-D).

*Note:*

In addition to the mouth fixation, the tube face mask used in this protocol also participates in the anesthesia maintenance.

*Skull exposure:*

**Fig 2:**
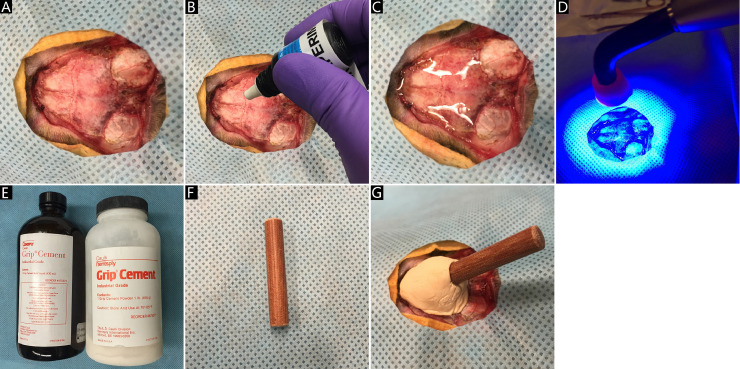
Skull exposure and skull cap establishment. A: the parietal bones and the latter half of frontal bone exposure with the help of a scalpel; B: application of the universal dental adhesive; C: a layer of universal dental adhesive on the skull surface exposed; D: the UV curing light and the solidification of universal dental adhesive; E: the grip cement powder (right) and grip cement bulk liquid (left); F: a wooden rod; G: the skull cap with a wooden rod imbedded, produced by the sticky fluid compound of the dental resin.

9. 0.5% iodophor is applied to the exposed skin on the top of the animal head to achieve the purpose of disinfection.

10. 0.5% xylocaine as additional local anesthesia is applied to the local soft tissues of the operation field just on the top of skull.

8. The skin and tissues on the top of parietal bones and the latter half of frontal bone are all removed carefully with the aid of a scalpel (Fig 2A).

9. Any bleeding in the exposed field is stopped strictly by an electrocautery.

#### Skull cap establishment

10. After completely well exposure, the top surfaces of parietal bones and the latter half of the frontal bone are dried as much as possible.

11. A layer of universal dental adhesive (light-cure universal dental adhesive, model 35,108, OptiBond XTR selfetch, USA) enhancing marginal integrity and reducing microleakage for long-lasting restorations is evenly covered on the surface of these bones (Fig 2B).

13. The UV curing light (Maxima LED curing light, RU1200, USA) is used for about 60–80 s to solidify the dental adhesive (Fig 2C-D).

14. The grip cement powder (CAULK/DENTSPLY, 675,571, USA) is well mixed with grip cement bulk liquid (CAULK/DENTSPLY, 675,572, USA) to form a sticky fluid compound (Fig 2E).

16. A wooden rod of about 8 mm in diameter is imbedded in the skull cap before dental resin curing (Fig 2F).

15. When the layer of dental adhesive strongly grips the skull, the sticky fluid compound of the dental resin is straightly applied on the adhesive to establish a skull cap (Fig 2G).

*Note:*

The curing time for the dental resin skull cap is about 10 min generally. Therefore, wooden rod should be imbedded in the skull cap immediately after the skull cap is built. Additionally, the wooden rod will produce a rigid bridge from skull to stereotaxic chair not only to provide stable head fixation but also to avoid any phase difference between recording electrode and animal head.

#### The vestibular nerve exposure

**Fig 3:**
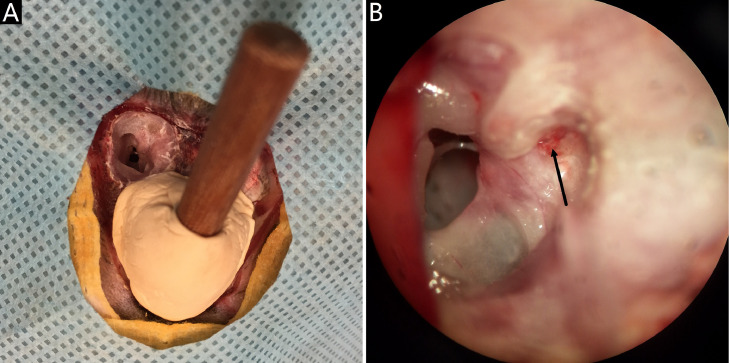
Exposure of the superior vestibular nerve. A: exposure of the inner ear by opening the top wall of the tympanic cavity; B: a small fenestra (black arrow) at the inside of confluence of the anterior semicircular canal and the horizontal semicircular canal for the exposure of the superior vestibular nerve bundle.

16. Additional local anesthesia (Xylocaine, 0.5%) is applied to the local soft tissues above the tympanic cavity (mastoid bulla) of the temporal bone (right side).

17. The skin and subcutaneous tissue covering the tympanic cavity are removed to expose the bone surface.

18. The tympanic cavity is opened from its top wall to expose the anterior semicircular canal and its ampulla, the horizontal semicircular canal and its ampulla, the facial nerve canal and petrous part of temporal bone (Fig 3A).

19. A small fenestra (0.5 mm × 0.5 mm) is dilled at the inside of confluence of the anterior semicircular canal and the horizontal semicircular canal, from where the superior vestibular nerve bundle could be exposed without any interference with brain structures (Fig 3B).

*Note:*

The anatomical features of the tympanic cavity of chinchilla are completely different from that of humans and other mammals. The superior tympanic cavities in chinchilla are quite large. Thus, the top walls of the tympanic cavities are located on both sides of the middle of the posterior skull top. In addition, the bone wall at the top of the superior tympanic cavity is very thin, which leads to the superior vestibular nerve canal is easy to access through this surgical approach described above.

#### Glass microelectrodes setup and single unit recording setup

**Fig 4:**
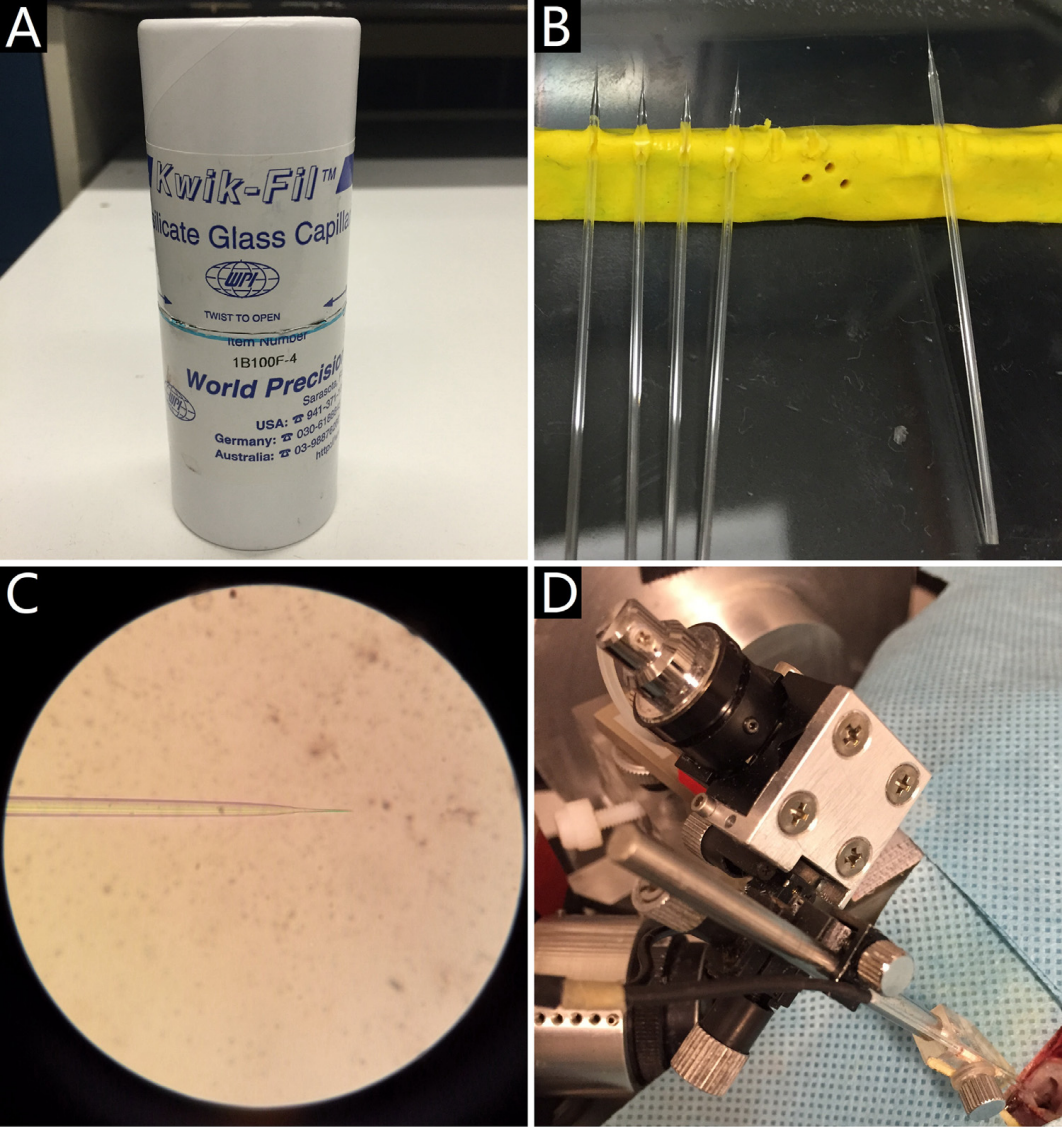
Glass microelectrode setup for single unit recording. A: glass micropipettes; B: glass microelectrodes pulled by glass micropipettes; C: a glass microelectrode filled with 3 mol/L NaCl solution to achieve suitable impedance; D: the glass microelectrode held in the position upon the fenestra by a three-dimensional manipulator fixed on a hydraulic Microdrive.

20. Glass micropipettes (WPI, model M1B100F-4, United Kingdom) (Fig 4A) with dust free are pulled to produce glass microelectrodes (Fig 4B) by microelectrode puller (P-30).

21. Glass microelectrodes are afterward filled with 3 mol/L NaCl solution to achieve 20–40 mΩ impedance (Fig 4C), measured by impedance meters (SYS-OMEGAZ).

22. Then the glass microelectrode is held in position upon the fenestra using a three-dimensional manipulator (You, model US-3F, Japan) fixed on a hydraulic Microdrive (Narishige International USA, models MO-22, USA) (Fig 4D) rigidly built in the gimbaled superstructural platform.

23. The reference electrode produced by the silver wire (AG10T) has a needle shape, which directly penetrates the neck skin and subcutaneous tissue into the neck muscles straightly.

24. The glass microelectrode is connected to the headstage amplifier (resistance 1.1 to 10 K).

25. The headstage amplifier is connected to the extracellular amplifier system (Dagan, model 2400A, USA) at the gains from 500 to 5000 and the band-pass filter from 300 to 3000 Hz.

26. Extracellular preamplifier system is connected to the CED Spike2 neural signal acquisition [4,5].

*Note:*

It should be pointed out that only the superior vestibular nerve was recorded by single unit recording in the present study because of the anatomical features of the chinchilla.

#### Animal head fixation two

**Fig 5:**
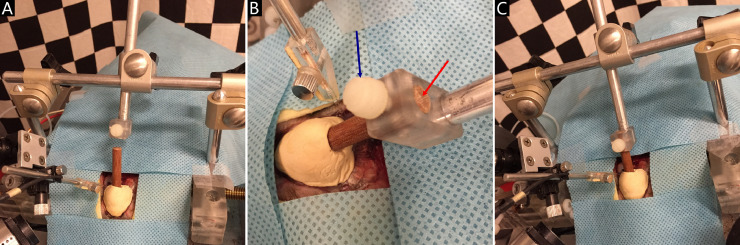
Animal head fixation based on the skull cap. A: a supporting frame installed on the stereotaxic chair; B: the fixation channel (red arrow) and the screw (blue arrow); C: the rigid bridge connection between the animal skull and the stereotaxic chair.

27. The animal stereotaxic chair for preliminary animal head fixation is fixed on the gimbaled superstructural platform atop an earth-vertical rotating table driven by the Servo-controlled rate motor (D083M-22–1310). A supporting frame designed for large-area skull contact fixation is installed on the stereotaxic chair, which is around the animal head (Fig 5A).

28. The wooden rod imbedded in the skull cap is inserted into the fixation channel of the supporting frame (Fig 5B).

29. Then the screw on the fixation channel wall is tightened to make sure that the supporting frame grips the wooden rod imbedded in the skull cap, which provides a rigid bridge between the animal skull and the stereotaxic chair (Fig 5C).

*Note:*

If the supporting frame is disconnected from the rod, the head fixed by the ear bars and tube face mask is similar to the head fixation generally used in previous studies [1], [2], [3]. It should be emphasized that the animal lays on the animal stereotaxic chair, where the animal body is adjusted to ensure that the longitudinal axis of the animal body and the head line connecting centers of left and right external auditory canals both run through the rotation axis. Therefore, the chinchilla head rotation in the present study is whole-body head rotation, not the head rotation relative to the body.

#### Single unit recording during rotational stimulation with large amplitude

**Fig 6:**
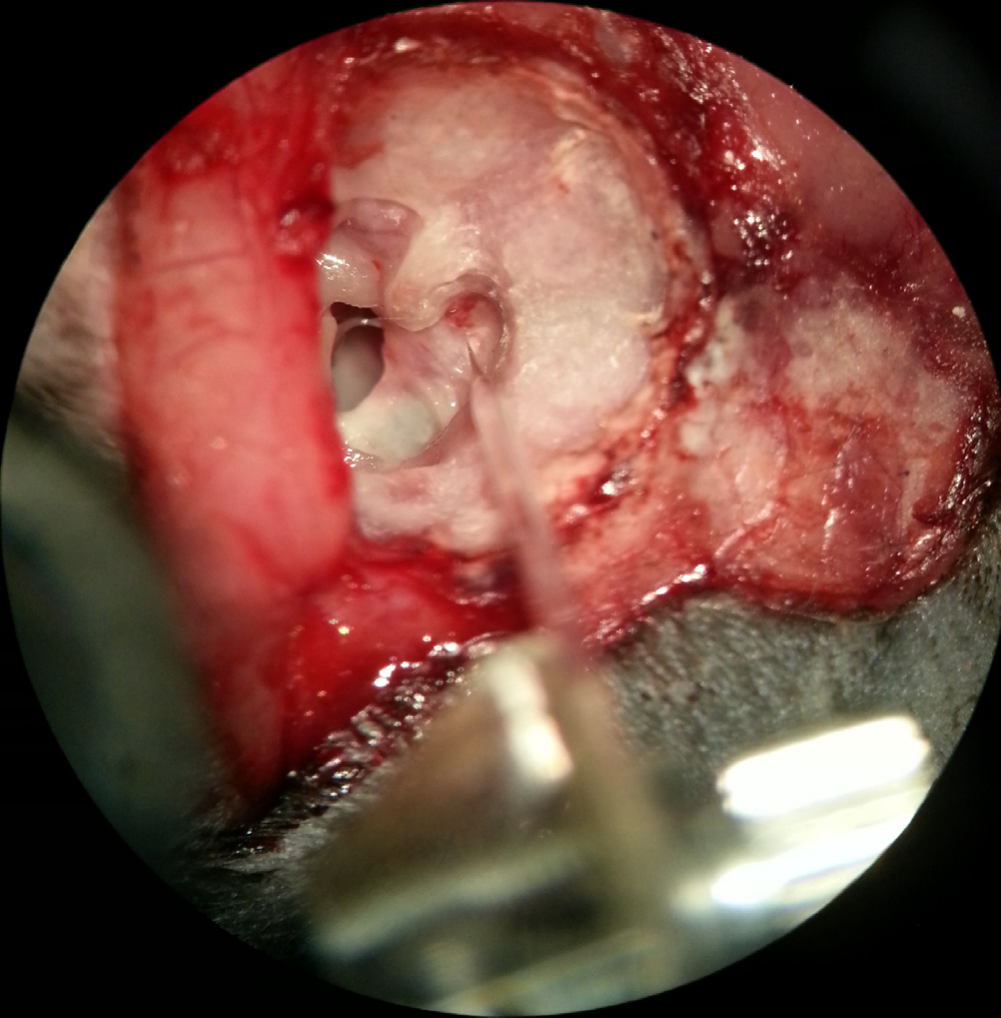
A glass microelectrode accessing the vestibular nerve tissue through the fenestra on the superior wall of internal auditory canal for single unit recording.

**Fig 7:**
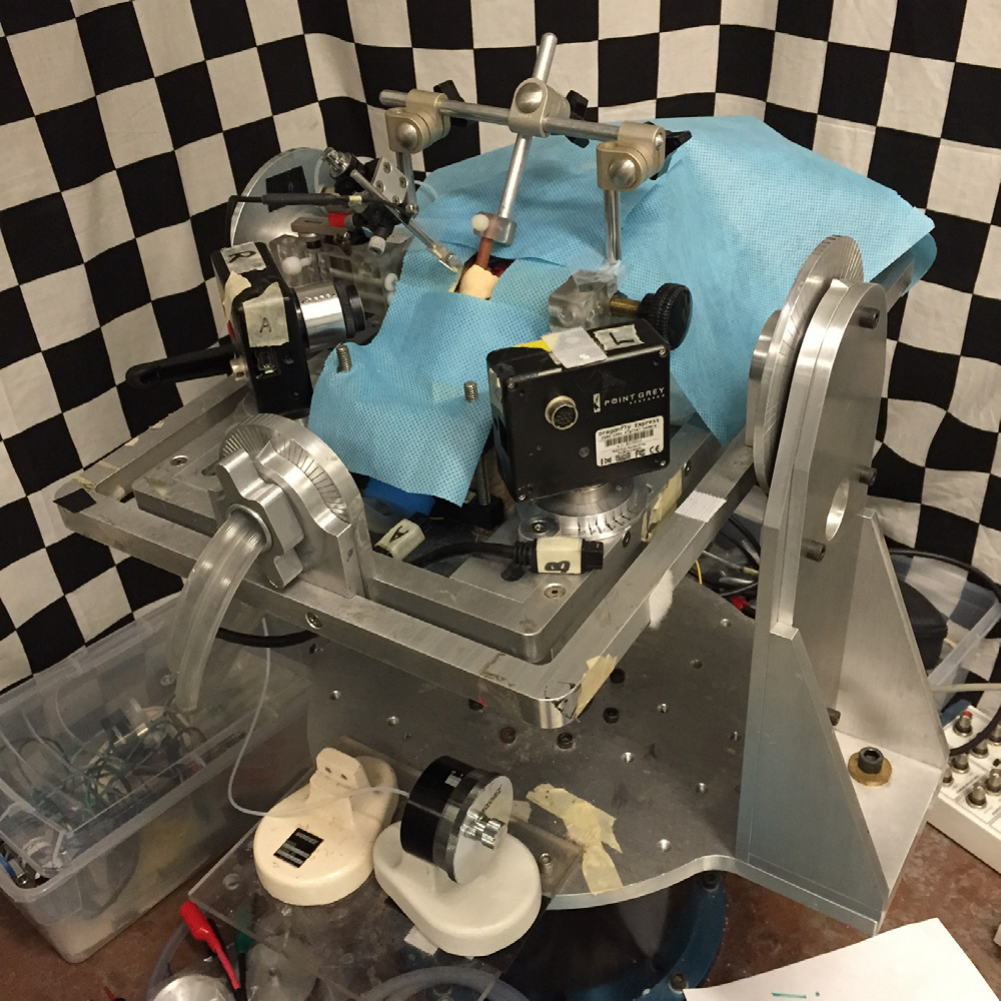
An earth-vertical rotating table driven by the servo-controlled rate motor supporting the sinusoidal rotation stimuli.

31. After single unit recording setup and head fixation, the glass electrode driven by the hydraulic Microdrive is carefully inserted into the vestibular nerve tissue through the dura exposed in the fenestra (Fig 6).

32. The hydraulic Microdrive should be slowly advanced until the glass electrode isolates a single nerve fiber or neuron cell body and identifies the extra axonal activity of neuron (spikes).

33. Once a neuron is well isolated, its innervation on the vestibule is immediately identified through monitoring the neural dynamic in response to a combination of stimuli [6]. Only semicircular canal afferent neurons will be collected to conduct the further experiment in this article.

34. Then the prefer plane of the semicircular canal afferent neuron is brought into the rotation plane of the rotating table by adjusting the gimbaled platform [4,5].

35. Finally, the 0.2 Hz sinusoidal rotation with sequentially increased amplitude supported by the Servo-controlled rate motor (D083M-22–1310) is applied to stimulate the semicircular canal afferent neuron (Fig 7). The amplitudes used in this article are 60, 80, 100, 120, 150, 180, 250, 300 deg/s. The sinusoidal rotation is repeated 5 cycles at each amplitude. The neural response dynamic during the stimulation will be continuously recorded (Fig 7).

## Results

**Fig 8:**
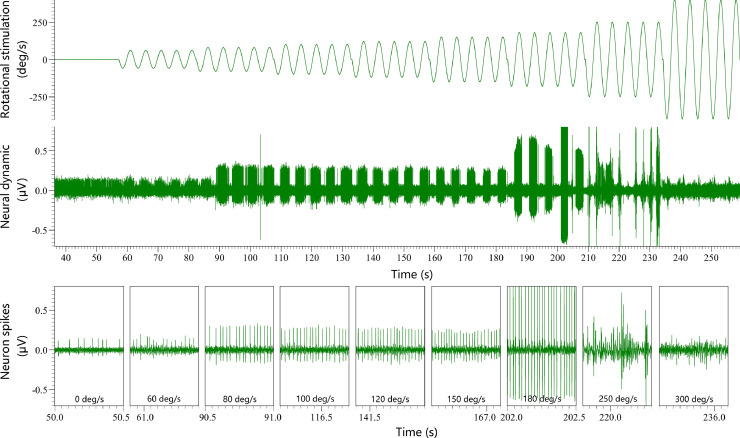
The neural dynamic of a semicircular canal afferent neuron (CV* = 0.104) recorded by single unit recording supported by the head fixation based on two ear bars and a tube face mask.

**Fig 9:**
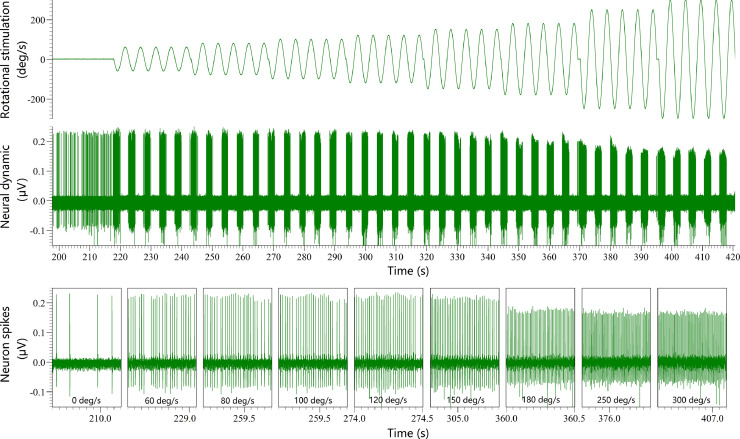
The neural dynamic of a semicircular canal afferent neuron (CV* = 0.192) recorded by single unit recording supported by the head fixation based on skull cap.

According to this protocol, we have successfully conducted the head fixation based on skull cap for single unit recording. In order to confirm the advantages of this novel head fixation, we have recorded the neural dynamics of semicircular canal afferent neurons in response to motion stimulation with different magnitudes through single unit recording, where the mechanical stability was provided by head fixation based on skull cap and head fixation based on two ear bars and tube face mask respectively. There are some examples of neural response dynamics recorded by the single unit recording supported by head fixation based on two ear bars and tube face mask (Fig 8) and based on skull cap (Fig 9), separately. It can be easily observed that the noise was small and the spike trains were stable during the whole process of single unit recording based on the head fixation of skull cap (Fig 9). However, the spike trains were very unstable during the single unit recording based on the head fixation of two ear bars and a tube face mask (Fig 8). The spike trains were lost and replaced by huge noise when the amplitude was larger than 180 deg/s (Fig 8). Further, our recent study demonstrated that the success rate of single unit recording in semicircular canal afferent neuron population was significantly improved by the head fixation based on skull cap when the amplitude of sinusoidal rotation stimuli was larger than 100 deg/s (Chi-square test, *P* < 0.001 when amplitude was larger than 100 deg/s) [5]. These results above confirmed that this protocol can significantly improve the mechanical stability for single unit recording and laid a foundation to understand the vestibular system in depth.

## Declaration of Competing Interest

The authors declare that they have no known competing financial interests or personal relationships that could have appeared to influence the work reported in this paper.
